# O-GlcNAc Modification Alters the Chaperone Activity of HSP27 Charcot-Marie-Tooth Type 2 (CMT2) Variants in a Mutation-Selective Fashion

**DOI:** 10.1021/acschembio.3c00292

**Published:** 2023-08-04

**Authors:** Stuart P. Moon, Binyou Wang, Benjamin S. Ahn, Andrew H. Ryu, Eldon R. Hard, Afraah Javed, Matthew R. Pratt

**Affiliations:** ^†^Departments of Chemistry and ^‡^Biological Sciences, University of Southern California, Los Angeles, California 90089, United States

## Abstract

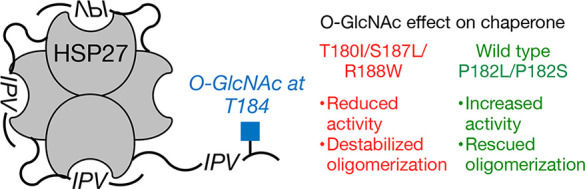

Increased O-GlcNAc is a common feature of cellular stress, and the upregulation of this dynamic modification is associated with improved survival under these conditions. Likewise, the heat shock proteins are also increased under stress and prevent protein misfolding and aggregation. We previously linked these two phenomena by demonstrating that O-GlcNAc directly increases the chaperone of certain small heat shock proteins, including HSP27. Here, we examine this linkage further by exploring the potential function of O-GlcNAc on mutants of HSP27 that cause a heritable neuropathy called Charcot-Marie-Tooth type 2 (CMT2) disease. Using synthetic protein chemistry, we prepared five of these mutants bearing an O-GlcNAc at the major site of modification. Upon subsequent biochemical analysis of these proteins, we found that O-GlcNAc has different effects, depending on the location of the individual mutants. We believe that this has important implications for O-GlcNAc and other PTMs in the context of polymorphisms or diseases with high levels of protein mutation.

Small heat shock proteins (sHSPs) are a vital class of ATP-independent chaperones capable of mediating the folding and proteostasis of their clients.^1,2^ sHSPs bind unfolded and partially misfolded proteins in their environments in order to shield them from forming aggregation-competent structures or to halt further misfolding and allow for clearance of the client by the appropriate cellular machinery.^3^ Members of this family contain a central, conserved α-crystallin domain (ACD) that contains a hydrophobic cleft for client interactions (Figure 1a). The variable N-terminal domains of these proteins are important for their ability to form large, dynamic oligomers, the size and organization of which tune their chaperone activity. Finally, a number of sHSPs (HSP27, αA-, and αB-crystallin (αAC and αBC)) contain an IXI/V motif within their C-terminal domains. This hydrophobic region of the protein has been shown to bind reversibly to the ACD domains of neighboring sHSPs within the chaperone oligomer, and thereby both inhibits the binding of clients to that monomer and alters the size and structure of the oligomer itself.^4−7^ Therefore, this autoregulatory interaction is critically important to protein function and has implications in diseases related to decreased molecular chaperone activity.

**Figure 1:**
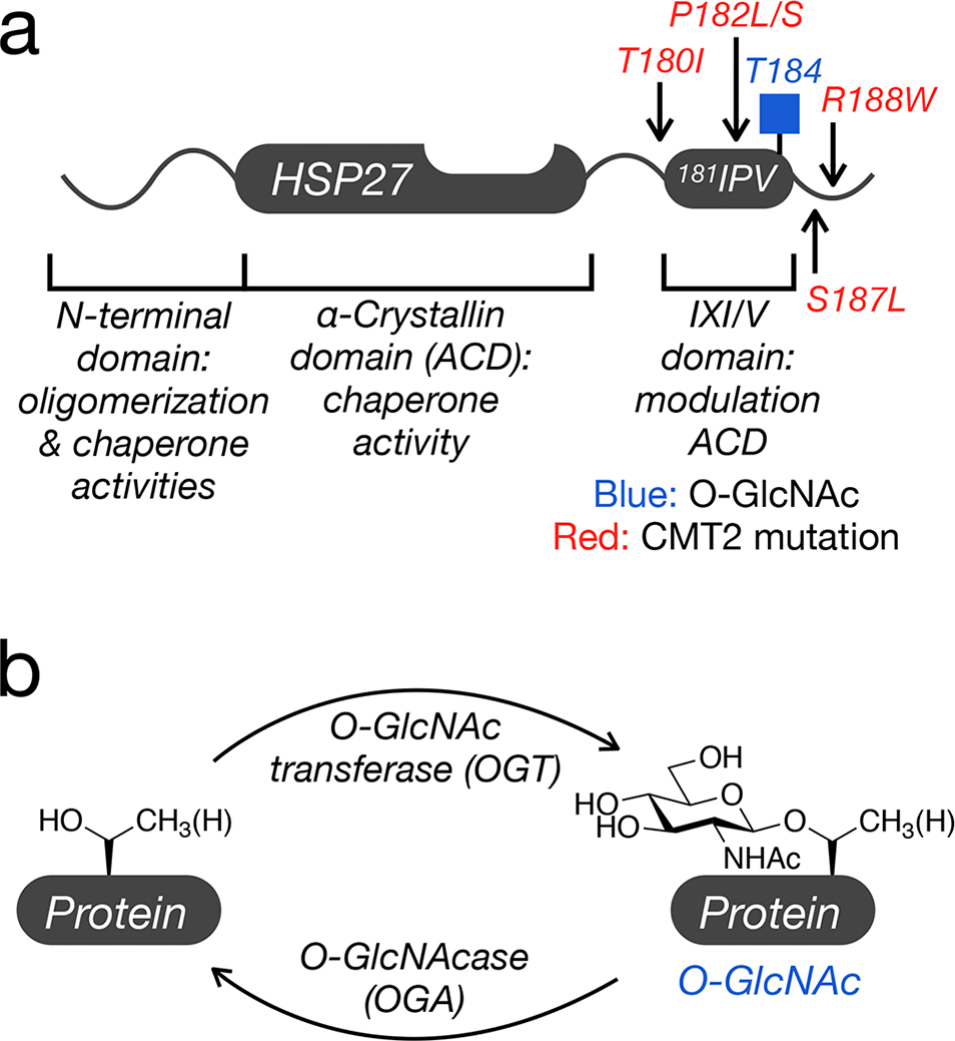
HSP27 and O-GlcNAc modification. (a) HSP27 has three important domains: an N-terminal domain that facilitates oligomerization, a central α-crystallin domain (ACD) that binds many clients, and a C-terminal IXI/V domain that can bind back to the ACD. Several mutations that cause Charcot-Marie-Tooth type 2 (CMT2) disease as well as O-GlcNAc are located near the IXI/V. (b) O-GlcNAc is the dynamic glycosylation of serine/threonine residues on intracellular proteins.

Charcot-Marie-Tooth type 2 (CMT2) disease is a hereditary neuromuscular disease characterized by the loss of the structure and function of peripheral neurons within the extremities. It is the most common inheritable peripheral neuropathy, and while not lethal, it results in disability and can progress to cause severe nerve pain. Several point mutations of HSP27 have been identified in CMT2 patients, and these mutations have been shown to impart CMT2-like symptoms in transgenic mouse models, implicating the dysregulation of this protein as a driver of disease pathology.^8,9^ Interestingly, several of these point mutations cluster around the conserved IXI/V motif in the protein’s C-terminus (residues 181–183, IPV; Figure 1a).^10−14^ Several of the following mutations (T180I, P182L, P182S, S187L, and R188W) have been shown to impact the oligomerization and/or the chaperone activity of the protein against a number of clients in *in vitro* studies,^15,16^ presumably because of an imbalance in the reversibility of the ACD–IPV interaction. More specifically, these previous studies show that mutation at T180I and at R188W increases the protein’s thermal stability and does not significantly alter its oligomerization; however, it results in decreased chaperone activity toward amorphous aggregation. P182L and P182S mutations also decrease HSP27’s chaperone activity and are associated with the formation of oligomers that are ∼30× and ∼45× larger than WT oligomers, respectively. The S187L variant has not been characterized biochemically; however it has been shown to behave similarly to P182L/S in *in vivo* studies, forming large oligomer structures.

Intriguingly, HSP27, αAC, and αBC contain a conserved site of O-GlcNAcylation directly adjacent to their IPV sequences (at T184 in HSP27, Figure 1a).^17−20^ O-GlcNAcylation is the dynamic addition of a single monomer of *N*-acetylglucosamine (GlcNAc) to the side chain hydroxyls of serine and threonine residues of intracellular proteins, and it is a required post-translational modification (PTM) for development and survival in mammals and insects (Figure 1b).^21,22^ The global levels of PTM have been shown to correspond to metabolic, homeostatic, and disease states of the cell. The moiety imparts site-specific effects on its substrates via myriad, context-dependent mechanisms, such as by disrupting protein–protein interactions due to its relatively large, hydrophilic structure.^23−26^ Because of this, we previously used protein chemistry to build site-specifically O-GlcNAcylated variants of sHSPs to test the PTM’s functions *in vitro*.^25,27^ We showed that the modification resulted in chaperones that were significantly better at preventing protein aggregation than their unmodified counterparts. We also used a variety of biophysical experiments to demonstrate that the presence of O-GlcNAc alters the size and makeup of HSP27 oligomers and does indeed disrupt the ACD-IXI/V interaction. The exact stoichiometry of O-GlcNAc at this site on HSP27 is currently unknown, but the levels of O-GlcNAc at the same conserved site in the related sHSP α-crystallin A has been measured at anywhere from 2 to 50% depending on the detection method.^17^ Furthermore, this PTM acts in a “turn-on” fashion in the wild-type chaperone, suggesting that even a small amount of modification could have a biological effect.

While there is no evidence to indicate a link between dysregulation of cellular O-GlcNAc levels and CMT2, the effects of the O*-*GlcNAc moiety on the ACD-IPV interaction increased the chaperone activity of wild-type HSP27, so we hypothesized that they might outcompete the antichaperone effects imparted by the CMT2 point mutations and thus rescue chaperone activity. We test this possibility here by using protein chemistry to site-specifically install O-GlcNAc at HSP27’s conserved modification site in conjunction with each of the five CMT2 point mutations. These semisynthetic variants of the protein were then compared to their unmodified, mutant counterparts via both amyloid β (Aβ) aggregation assay and size-exclusion chromatography–multiangle light scattering (SEC-MALS) to test the PTMs functional consequences on each mutant’s chaperone activity and oligomerization propensity, respectively. We found that O-GlcNAc has different effects depending on the specific CMT2 mutation. Consistent with our original hypothesis, O-GlcNAc modification rescued the chaperone activity and oligomerization state of mutants directly within the IPV: P182L and P182S. However, the modification was detrimental to the activity of the other HSP27 mutants located further from the IPV sequence. Additionally, O-GlcNAc modification of these mutant resulted in the formation of heterogeneous, low-molecular-weight protein species, consistent with their reduced chaperone activity. While more complicated than originally anticipated, our results support an overall model where HSP27 activity is highly dependent on interactions mediated by the IPV-containing C-terminal tail and the formation of properly folded oligomers. They also suggest that PTMs like O-GlcNAc could have opposing consequences for human disease depending on the context of the underlying protein sequence, a cross-talk feature that is largely overlooked as most studies consider either mutations or PTMs in isolation.

The functional analysis of site-specific PTMs ideally involves homogeneously modified protein, which can be difficult for many modifications, especially O-GlcNAc. For example, enzymatic modification of recombinant proteins with the O-GlcNAc transferase typically results in a substoichiometric mixture of modified sites that can be difficult to purify. Additionally, unlike other PTMs including phosphorylation and acetylation, O-GlcNAc cannot be installed using genetic codon expansion or reliably mimicked by any of the 20 natural amino acids. To overcome these limitations, we have exploited protein synthesis through the use of ligations,^28,29^ specifically the native chemical ligation (NCL) reaction.^30^ Briefly, NCL refers to the selective reaction between protein thioesters and N-terminal cysteines that results in a native amide bond. Using NCL and its extension, expressed protein ligation (EPL),^31^ proteins can be assembled from multiple recombinant and synthetic fragments for the installation of site-specific modifications like O-GlcNAc.

In our synthetic scheme (Figure 2), we used recombinant expression and intein technology to generate an N-terminal (2–172) fragment of HSP27 bearing a C-terminal thioester (**1**). In this case, we chose to mutate the lone native cysteine (C137) to an alanine for ease of synthesis and purification. Importantly, this cysteine has been shown to not play a critical role in the antiaggregation activity of HSP27,^32^ although it can be important in other contexts such as oxidative sensing.^33^ In parallel, we used solid-phase peptide synthesis (SPPS) with an O-GlcNAc threonine^34^ to construct C-terminal fragments (residues 173–205, **2a**–**2e**) bearing three critical features: (1) native alanine residue 173 was converted to a cysteine required for the ligation reaction, (2) one of the five CMT2 mutations of interest, and (3) an O-GlcNAc residue at the major site of modification, threonine 184. Subsequent individual ligation reactions between these peptides and C-terminal thioester **2** gave the corresponding O-GlcNAc-modified HSP27 mutants (**3a**–**3e**). Unfortunately, we were unable to separate any of the full-length proteins **3a**–**3e** from N-terminal fragment **1** using reverse-phase high performance liquid chromatography (RP-HPLC), but we chose to proceed with the synthesis using the crude ligation-reaction mixture.

**Figure 2:**
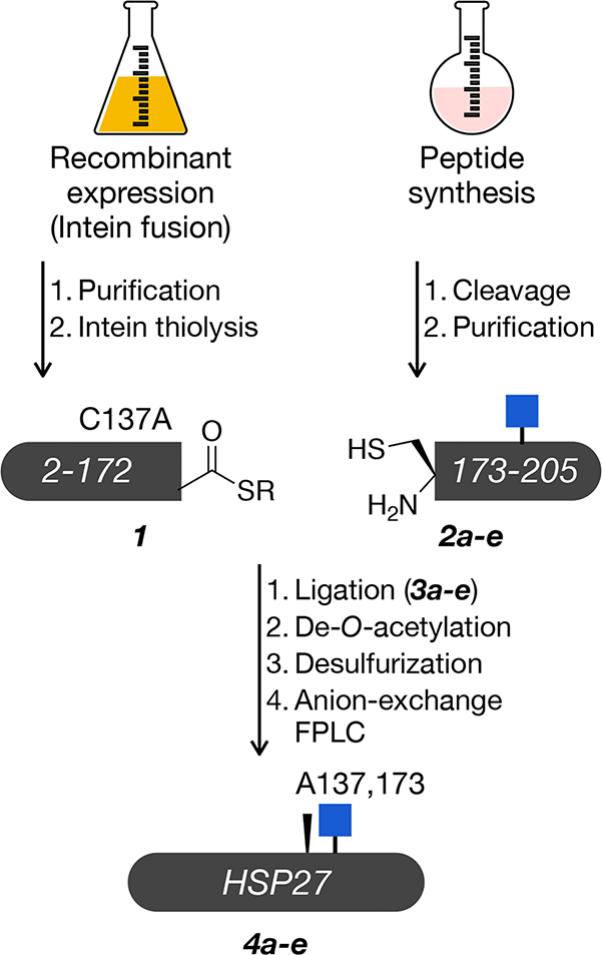
Synthesis of the O-GlcNAc modified HSP27 mutants. The majority of the protein was produced recombinantly as thioester **1** as an intein fusion followed by thiolysis. The O-GlcNAc modification at T184 (gT184) and individual CMT2 mutations were incorporated by solid-phase peptide synthesis (**2a**–**2e**). Full-length proteins (**3a**–**3e**) were generated by ligation, followed by deprotection/desulfurization and purification by anion exchange to yield the final products (**4a**–**4e**).

Next, we removed the *O*-acetyl protecting groups on the GlcNAc residue and performed radical desulfurization to convert cysteine 173 back to the native alanine residue, yielding the final protein products (**4a**–**4e**). This one-pot strategy involved several buffer exchange steps in which the products were subjected to spin filtration with 10 kDa molecular weight cutoff filters, effectively removing unreacted peptides **2a**–**2e** (∼3.8 kDa). Again, we were unable to separate **1** from **4a**–**4e** by RP-HPLC. We therefore turned instead to anion exchange fast protein liquid chromatography (FPLC), which afforded good separation (Figure S1). Fractions bearing purified **4a**–**4e** were pooled and dialyzed slowly into DPBS to remove denaturants and promote refolding. Importantly, this synthetic route enabled us to perform the ligation, deprotection, and desulfurization steps in one pot by simply exchanging the reaction buffers with a spin column. In this way, we successfully constructed, purified, and refolded a panel of semisynthetic HSP27 mutants bearing a site-specific O-GlcNAc modification. The final protein products were characterized by RP-HPLC and electrospray mass-spectrometry (ESI-MS; Figure S2). Finally, we produced the corresponding nonglycosyated proteins by recombinant expression, purified them by RP-HPLC, and characterized the purified products by RP-HPLC and ESI-MS (Figure S3). These proteins were then refolded and analyzed by circular dichroism spectroscopy (Figure S4). Consistent with previous reports, the HSP27 CD spectra contain a wide minimum around 215 nm corresponding to the β-sandwich-like fold of the ACD.^35^ All of the CMT2 mutants and most of their O-GlcNAc modified variants also display a similar minimum, suggesting that they are similarly folded at a secondary and tertiary level. Notably, the modified versions of T180I and R188W also show a minimum below 210 nm, indicating the potential for partially poorly folded protein.

With our range of proteins in hand, we moved to determine the consequences of the O-GlcNAc modification. HSP27 chaperone activity is typically measured using two broad types of protein aggregation assays. In one, HSP27 is added to folded proteins, followed by the induction of thermal denaturation and visualization of subsequent protein and amorphous aggregation/precipitation using absorption at 400 nm. Alternatively, HSP27 can be added to a protein or peptide that undergoes amyloid aggregation, such as Aβ or insulin, which can be measured using an amyloid sensitive dye such as Thioflavin T (ThT). In our case, we were concerned about the reliability of the first type of experiment, as the CMT2 mutants can cause HSP27 to be thermally destabilized itself, and the effect of O-GlcNAc on this process is unknown. Therefore, we chose to analyze the chaperone activities of our proteins by examining the inhibition of amyloid-β (Aβ1–42) at 37 °C. Another complicating feature of these assays is the fact that HSP27 affects the aggregation process ratiometrically, where the addition of more chaperones increases the onset time or delay in Aβ1–42 aggregation. Thus, we needed to first establish a ratio of HSP27 to Aβ1–42 for each mutant, where we could measure any effect of O-GlcNAc. In other words, the amount of HSP27 mutant should be sufficient to slow aggregation at a level less than that of the wild-type chaperone but still significantly compared to that of no chaperone at all. We first mixed Aβ1–42 with individual HSP27 proteins (wild-type or mutants) at ratios of 15, 12.5, or 10 to 1 of Aβ1–42:HSP27 and monitored aggregation by ThT (Figure S4a). For the T180I, S187L, and R188W mutants, a ratio of 15:1 provided good separation in the chaperone activities compared to both the wild-type protein and no chaperone aggregation conditions. However, for the P182L and P182S mutants, even the lowest ratio of 10:1 did not show notable chaperone activity. We then mixed Aβ1–42 individually with these two mutants at even lower ratios of 7.5 or 5 to 1 and found that the 5:1 ratio was appropriate (Figure S4b).

With these initial characterization experiments completed, we then used the same assay to separately compare the CMT2 mutants to their O-GlcNAc modified counterparts (Figure 3a). We found that O-GlcNAc rescued the chaperone activity of the P182L and P182S mutants as we hypothesized. However, this effect was reversed for the T180I, S187L, and R188W mutants, where the O-GlcNAc diminished their chaperone activities. To quantify these results, we then measured the onset times as the time for the ThT signal to reach 2.75× the initial assay value (Figure 3b). In our experiments without HSP27 present, the ThT signal tends to decline as larger, amyloid structures form that are insoluble, a phenomenon seen by ourselves and others in the past. These larger aggregates do not form in the presence of our HSP27 variants, presumably due to the activity of the chaperones. Therefore, we chose to focus on the kinetics of aggregation (as opposed to the extent) for our quantification purposes. As expected, all of the chaperones increased the onset time of aggregation compared to Aβ1–42 alone, and we observed significant differences between the CMT2 mutants and their O-GlcNAc modified versions as described above.

**Figure 3:**
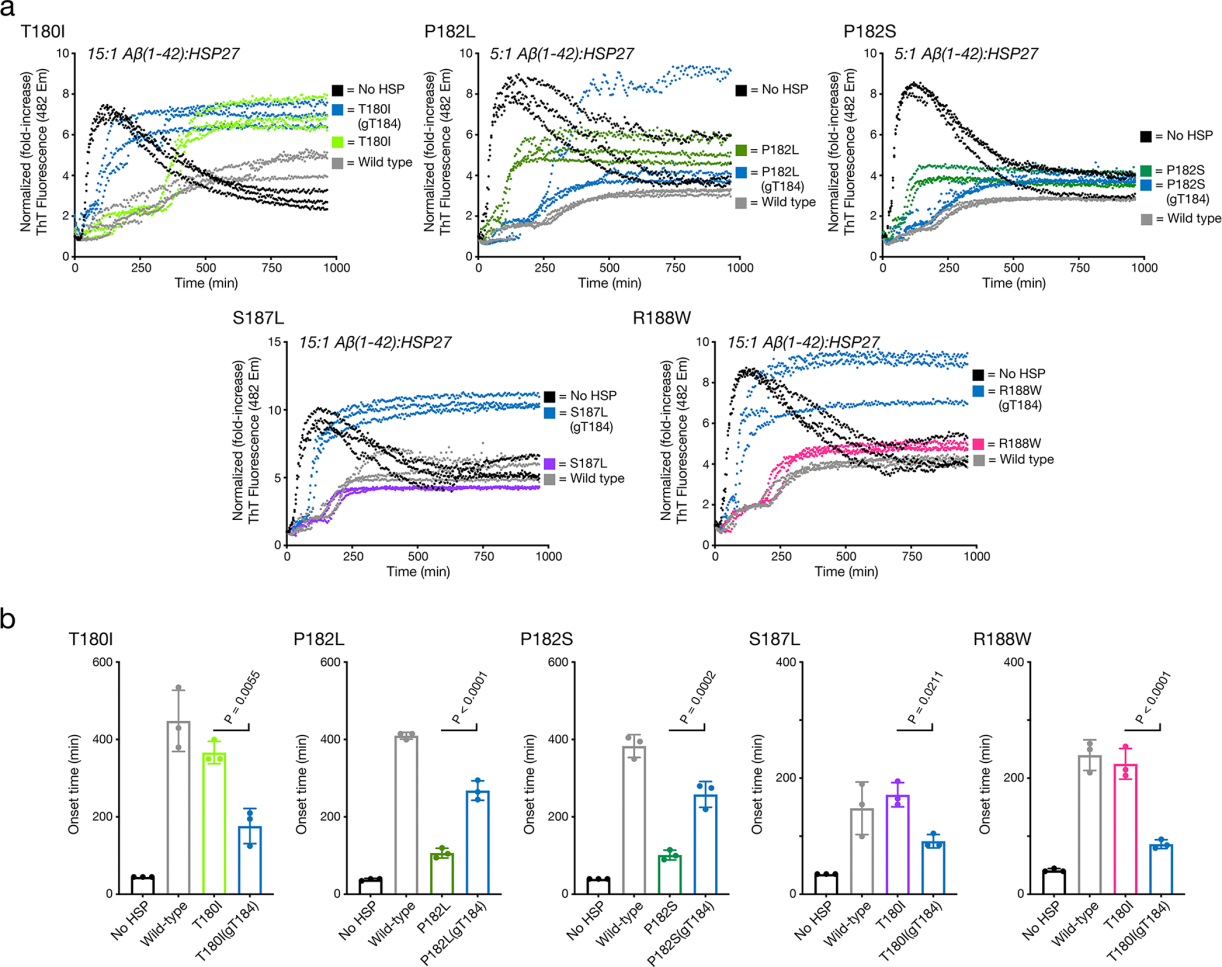
O-GlcNAc has mutation-dependent effects on HSP27 chaperone activity. (a) Aβ(1–42) alone (10 μM) or in the presence of the indicated ratios of HSP27 proteins was subjected to aggregation conditions (agitation at 37 °C in a plate reader). Every 5 min, ThT fluorescence (λ_ex_ = 450 nm and λ_em_ = 482 nm). (b) Onset times were obtained by measuring the time required for fluorescence to reach 2.75 times the initial reading. Onset time results are mean ± SEM of experimental replicates (*n* = 3). Statistical significance was determined using a one-way ANOVA test followed by Tukey’s test.

As a potential mechanism to explain these divergent results, we used size exclusion chromatography linked to a multiple angle light scattering detector (SEC-MALS) to examine the effects of the mutations and the O-GlcNAc on the HSP27 oligomeric structure (Figure 4). We first compared the CMT2 mutants to the wild-type chaperone and observed results consistent with previous reports.^15,16^ Specifically, P182L and P182S formed very large oligomers while T180I and R188W were similar in size to wild-type protein. S187L, which had not been previously characterized, showed a mixture of two peaks corresponding to a large oligomer like the P182 mutants and one similar to wild-type HSP27. Notably, the location of mutations with respect to the IPV sequence somewhat corresponds to their relative effects on the induction of a large oligomer with presumably reduced activity. This data also suggest that the T180I and R188W mutants are compromised in a way that may not be due to incorrect oligomer formation, which is not necessarily surprising given their distance from the IPV sequence. We then compared the CMT2 mutants to the O-GlcNAc variants (Figure 4). In agreement with the aggregation results, O-GlcNAc modification of the P182L and P182S oligomers resulted in a shift in the SEC trace and the formation of a major oligomer of size similar to that of the wild-type protein. In the cases of T180I, S187L, and R188W, the oligomer form of O-GlcNAc appears to destabilize near the wild-type size, resulting in major protein peaks at lower molecular weight. Notably, the MALS traces of these species are quite heterogeneous, suggesting the possibility of multiple incorrectly folded smaller species. Combined with our previous results on O-GlcNAc modification of wild-type HSP27 by O-GlcNAc, these results suggest a model whereby O-GlcNAc can rescue the CMT2 mutants that form large oligomers resulting from incorrect, direct ACD-IPV interactions but destabilizes the other mutants by an as of yet unknown mechanism.

**Figure 4:**
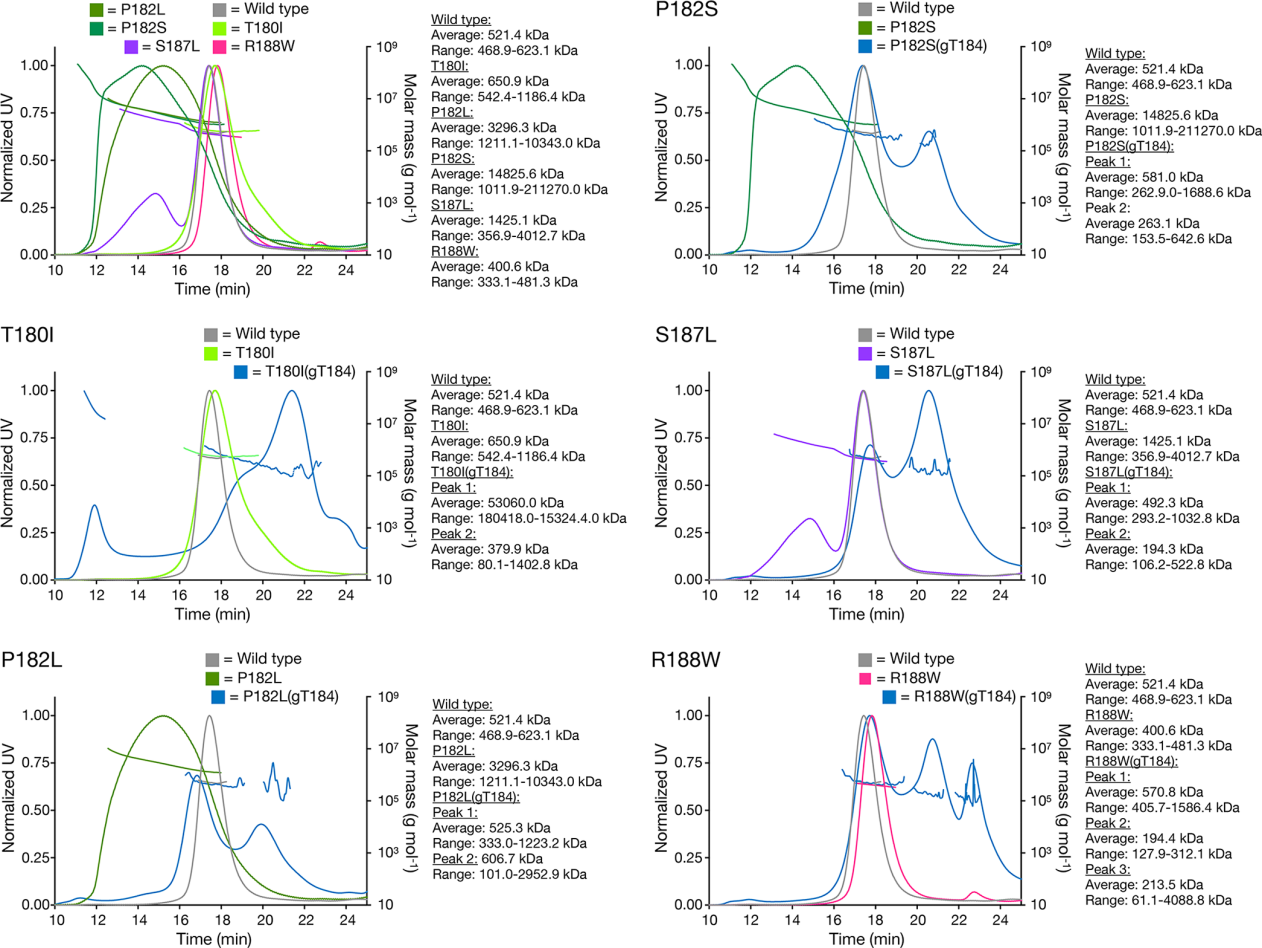
O-GlcNAc has mutation-dependent effects on the HSP27 oligomeric state. The indicated HSP27 proteins were analyzed by SEC-MALS. Average molecular weights and ranges measured by MALS are shown.

In summary, we used synthetic protein chemistry to prepare and characterize the effects of O-GlcNAc on CMT2-causing mutations of HSP27. We originally hypothesized that O-GlcNAc would dominate the biochemistry of the IXI/V domain of HSP27 where these mutations are located and thus rescue chaperone function. What we found was both more complicated and potentially more interesting. Specifically, O-GlcNAc was able to rescue the mutants that lie directly within HSP27’s IPV sequence but was detrimental to the activity of mutations located further away. This divergent activity appears to be a result of alterations in the oligomeric state of HSP27, as we determined using SEC-MALS. We speculate that this must be due to nonobvious interactions of the C-terminus outside of the ACD or through altering the folding process but does not have a molecular mechanism that cleanly explains all of our results. In the cases of T180I and R188W, O-GlcNAc may at least partially act by preventing proper folding of the protein. It is unclear whether this necessarily would be the case for an already folded oligomer being subjected to O-GlcNAc modification at T184, but this is impossible to test with synthetic proteins. O-GlcNAc transferase displays some preference for proline at the −2 position to modified serine or threonine residues,^36^ making it likely that the P182L and P182S mutants will have less O-GlcNAc at T184 in cells. While the exact molecular mechanisms to explain these differences are still unknown and are a potential area of further exploration, our results demonstrate that O-GlcNAc, and presumably other PTMs, can have different biochemical effects depending on an individual protein’s sequence. We postulate that this may have important implications for the function of PTMs in other diseases with high mutational burdens like cancer.

## Methods

### General

All materials used were purchased commercially and used without purification. Aqueous solutions were prepared by using ultrapure water. Bacterial growth medium was prepared as directed by the manufacturer, and medium and cultures were handled aseptically. Antibiotic stocks were prepared at 1000× and stored at −20 °C. Protein concentration was determined by a BCA protein assay (Thermo Fisher Scientific). An Agilent Technologies 1200 Series HPLC outfitted with a diode array detector was used for analytical and semipreparative reverse phase (RP) HPLC using C4 or C18 Phenomenex columns from Agilent (buffer A, 0.1% trifluoroacetic acid in water; buffer B, 0.1% TFA, 90% acetonitrile in water). Mass spectrometry was performed using an Agilent HPLC/Q TOF MS/MS spectrometer. Anion-exchange fast protein liquid chromatography (FPLC) was performed on an Amersham Pharmacia Biotech KTA FPLC (UPC-900, P-920) using a HiTrap Q HP anion exchange column (Cytiva Life Sciences; buffer A, 4 M urea, 20 mM Bis-Tris, pH 7; buffer B, 4 M urea, 20 mM Bis-Tris, 500 mM NaCl, pH 7).

### Generation of Expression Plasmids

The preparation of WT HSP27 and HSP27 2–172 AvaE-6xHis intein fusion plasmids was described previously.^25^ These plasmids were mutated using an Agilent QuikChange Lightning kit following manufacturer protocols to incorporate any point mutations of interest (T180I, P182L, P182S, S187L, and R188W) as well as the C137A mutation.

### Recombinant Expression and Purification

BL21(DE3) *E. coli* were transformed with the appropriate plasmid via heat shock and grown on antibiotic-selective agar plates. Overgrowth cultures of these bacteria were grown by inoculating 50 mL flasks of LB (100 μg/mL ampicillin) with a single colony of these plates and incubating the broth at 37 °C overnight with orbital shaking at 250 rpm. These cultures were used to inoculate larger expression cultures (500 mL of Terrific broth, 100 μg/mL ampicillin), which were incubated as above until an OD_600_ of 0.6–0.8 was reached. Expression was induced with IPTG to a final concentration of 1 mM, and expression proceeded at 37 °C with shaking for 6 h. Bacteria were harvested by centrifugation at 6000*g*, 4 °C before storage at −20 °C. Cell pellets were resuspended in lysis buffer (6 M guanidine, 50 mM phosphate, 300 mM NaCl, 2 mM TCEP, 5 mM imidazole, 2 mM PMSF, pH 7.2) prior to lysis via tip sonication, followed by lysate clarification by centrifugation (10 000*g*, 20 min, 4 °C). The soluble portion of the lysate was incubated with pre-equilibrated Co-NTA agarose beads (GoldBio) for 1 h at 4 °C before flowthrough and stringent washing of the resin with wash buffer (4 M urea, 50 mM phosphate, 300 mM NaCl, 2 mM TCEP, 20 mM imidazole, pH 7.2). Finally, the protein fragment of interest was eluted with elution buffer (4 M urea, 50 mM phosphate, 300 mM NaCl, 2 mM TCEP, 250 mM imidazole, pH 7.2), and the elution fractions were pooled and buffer exchanged into transfer buffer (4 M urea, 1× DPBS, 2 mM TCEP, pH 7.2) using an Amicon Ultra-15 centrifugal filter (10 kDa MWCO). Intein fusions were then hydrolyzed or thiolyzed, depending on desired C-terminal functionality. Full-length proteins were hydrolyzed by the addition of 150 mM DTT and adjustment of pH to 8 followed by incubation at 37 °C for 2 days. The N-terminal fragment was instead thiolyzed by the addition of 250 mM MESNa (pH 7.2) followed by incubation at RT for 2 days. The products of these reactions were purified by semipreparative RP-HPLC and characterized by analytical RP-HPLC and Q-TOF MS. Purified full-length proteins were lyophilized at −80 °C and resuspended in FPLC buffer A prior to refolding as described below. Purified 2-172 thioester was lyophilized at −80 °C, aliquoted for ligations, and stored at −20 °C. Typical yields ranged from 2 to 5 mg per 3 L of TB.

### Solid-Phase Peptide Synthesis

SPPS was performed using standard Fmoc chemistry on a CEM Liberty Blue peptide synthesizer on a 0.05 mmol scale. Preloaded Wang resin was used as the solid support. Fmoc deprotection proceeded in two 5 min steps via 20% 4-methylpiperidine in DMF at 50 °C with bubbling. Coupling solutions were comprised of 5 equiv of the corresponding amino acid, 5 equiv of HBTU, and 10 equiv of DIPEA in DMF. Following deprotection and washing of the resin, couplings were performed at 50 °C for 10 min with bubbling, and each amino acid was double-coupled. GlcNAcylated threonine residues were added manually via double-coupling using 2 equiv of Fmoc-Thr(O-(Ac)_3_GlcNAc)-OPfp (prepared in-house as described previously)^34^ in DMF (overnight, RT). Following completion, peptides were globally deprotected and cleaved from the resin by RT incubation in 95% TFA, 2.5% water, and 2.5% triisopropylsilane for 4 h. Peptides were precipitated in cold ether before purification by semipreparative RP-HPLC and characterization by analytical RP-HPLC and Q-TOF MS. Purified peptides were lyophilized at −80 °C and aliquoted for ligations before storage at −20 °C.

### Protein Chemistry

Protein ligation was performed by resuspending 5 mg of 2–172 thioester and 5 mg (∼5 equiv) of the corresponding 173–205 (A173C, gT184) peptide to 2 mM thioester in degassed ligation buffer (6 M guanidine, 200 mM phosphate, 30 mM MPAA, 30 mM TCEP at pH 7–7.2) followed by RT incubation overnight with agitation. Following ligation, the O-acetyl protecting groups were removed from the sugar by the addition of hydrazine monohydrate to 5% v/v and incubation at 25 °C for 1 h. This reaction was quenched by the addition of glacial acetic acid to 5% (v/v), and the mixture was adjusted to pH 7. The deacetylated ligation mixture was buffer exchanged into degassed 6 M guanidine and 200 mM phosphate at pH 7 using Amicon Ultra-0.5 centrifugal filters (10 kDa MWCO) to sufficiently remove MPAA and deacetylation reagents. In the process, the mixture was concentrated to ∼100 μL, and 300 μL of degassed desulfurization buffer (6 M guanidine, 200 mM phosphate, 233.3 mM TCEP, 106.7 mM reduced glutathione, and 53.3 mM VA-044 initiator at pH 7) was used to transfer the protein to a fresh tube (175 mM TCEP, 80 mM glutathione, and 40 mM VA-044 final). Desulfurization proceeded for 1.75 h at 37 °C, and the mixture was buffer exchanged into degassed FPLC buffer A using Amicon Ultra-0.5 centrifugal filters (10 kDa MWCO). Progress through each reaction above was monitored via analytical RP-HPLC and Q-TOF MS. Yields through the final buffer exchange averaged ∼50%.

### FPLC and Refolding

Crude, buffer-exchanged desulfurization mixtures were submitted to anion exchange FPLC to remove any excess unreacted N-terminal fragment. Fractions were screened by SDS-PAGE and Coomassie staining to identify sufficiently pure fractions, and desired fractions were pooled and concentrated using Amicon Ultra-15 centrifugal filters (10 kDa MWCO). Purified, semisynthetic products (or recombinantly expressed full-length proteins) were then transferred to Tube-O–DIALYZERs (8 kDa MWCO, Medi; G-BIOSCIENCES) and diluted to ∼0.5 mg mL^–1^ prior to refolding by overnight dialysis against 1× DPBS and 2 mM DTT at 4 °C. Some mutants required a less aggressive refolding, which entailed separate overnight dialysis steps to exchange the protein from 4, to 2, to 1, to 0 M urea. Proteins were then concentrated using Amicon Ultra-0.5 centrifugal filters (10 kDa MWCO) and stored at 4 °C to generate working stocks of each protein species for subsequent functional assays. Yields through FPLC and refolding averaged ∼30% of input.

### Circular Dichroism (CD)

Refolded proteins were buffer exchanged into CD buffer (10 mM phosphate, pH 7.2) using 10 kDa MWCO spin filters. Samples were then diluted in additional CD buffer to reach a concentration of 0.1 mg mL^–1^. CD spectra were collected using a Jasco J-815 instrument fitted with a Peltier thermostated cell holder at 25 °C. CD data were obtained from 190 to 260 nm every 0.5 nm. Three acquisitions of each sample were obtained and averaged before a background correction was made to generate final spectra.

### Amyloid β Aggregation Assays

All aqueous solutions used in this assay, as well as the 96-well plate used, were kept on ice as much as possible. Lyophilized Aβ_1–42_ (Anaspec) was resuspended in 10% NH_4_OH to 1 mg mL^–1^, aliquoted, and lyophilized at −80 °C. The peptide was then resuspended in 1% NH_4_OH to 10 mg mL^–1^ and stored at −80 °C. Desired masses/volumes of the peptide were then diluted in aggregation buffer (degassed 1× DPBS, 2 mM DTT, pH 7.4) to 73.8 μM. Any preformed aggregates were cleared by centrifugation (20 000*g*, 20 min, 4 °C) and the supernatant was used as a concentrated Aβ stock. This stock was further diluted to 11 μM with more aggregation buffer, and 11 μM ThT (from a 20 mM stock in DMSO) was added just before the wells of the plate. HSP27 proteins were diluted from their refolded stocks into 30 μL volumes containing the desired molar equivalency of chaperone using 1× DPBS and 20 mM DTT. HSP27 proteins were then incubated at 45 °C for 20 min. The two protein solutions were combined (320 μL of Aβ and 30 μL of HSP27 or buffer) to give a final concentration of 10 μM Aβ, 10 μM ThT, and the desired concentration of HSP27 before aliquoting in triplicate (100 μL/well) in a 96-well plate. The plate was sealed with transparent film and incubated at 37 °C for 16 h with constant linear shaking at 1096 cpm in an Agilent BioTek Cytation plate reader. Over this 16 h, fluorescence of each well was read every 5 min (ƛ_ex_ = 450 nm, 9 nm bandpass. ƛ_em_ = 482 nm, 9 nm bandpass; read height, 8 nm).

### SEC-MALS Analysis of Oligomers

The oligomer size and distribution of refolded HSP27 proteins were analyzed via SEC-MALS using an Agilent 1200 HPLC system fitted with a Shodex 804 column and coupled to a DAWN HELEOS light scattering and rEX refractive index detector (Wyatt Technology Corporation). Samples were diluted to 1.5 mg mL^–1^ in 1× DPBS and 2 mM DTT, and 150 μg of material was analyzed in each injection (mobile phase, degassed 1× DPBS; flow rate, 0.5 mL/min).

## Supporting Information Available

Purification and characterization of all HSP27 proteins, analysis of protein folding by circular dichroism (CD), and characterization of CMT2 mutant chaperone activities (PDF)
