# Supplementary material for: O-GlcNAc Modification Alters the Chaperone Activity of HSP27 Charcot-Marie-Tooth Type 2 (CMT2) Variants in a Mutation-Selective Fashion
Source: ACS Chem Biol. 2023 Aug 4;18(8):1705–12. doi: 10.1021/acschembio.3c00292 (PMC10442854; doi:10.1021/acschembio.3c00292)
Supplement: Supplementary file 1 — cb3c00292_si_001.pdf [file cb3c00292_si_001.pdf]

Supporting Information

O-GlcNAc modification alters the chaperone activity of HSP27 Charcot-Marie-Tooth type 2 (CMT2) variants in a mutation-selective fashion

Stuart P. Moon[1], Binyou Wang[1], Benjamin S. Ahn[1], Andrew H. Ryu,[1] Eldon R. Hard,[1] Afraah Javed,[1] and Matthew R. Pratt[1,2,*]

[1]Departments of Chemistry and [2]Biological Sciences, University of Southern California, Los Angeles, California, 90089, United States
*Corresponding Author: Matthew R. Pratt
Email: matthew.pratt@usc.edu

Table of contents:

Figure S1. Purification of semisynthetic HSP27 proteins by anion exchange. **Page S2**
Figure S2. Characterization of O-GlcNAc modified proteins. **Page S2**
Figure S3. Characterization of unmodified proteins. **Page S3**
Figure S4. Circular dichroism (CD) spectra of all proteins **Page S4**
Figure S5. CMT2 mutants of HSP27 show different levels of compromised chaperone activity. **Page S5**

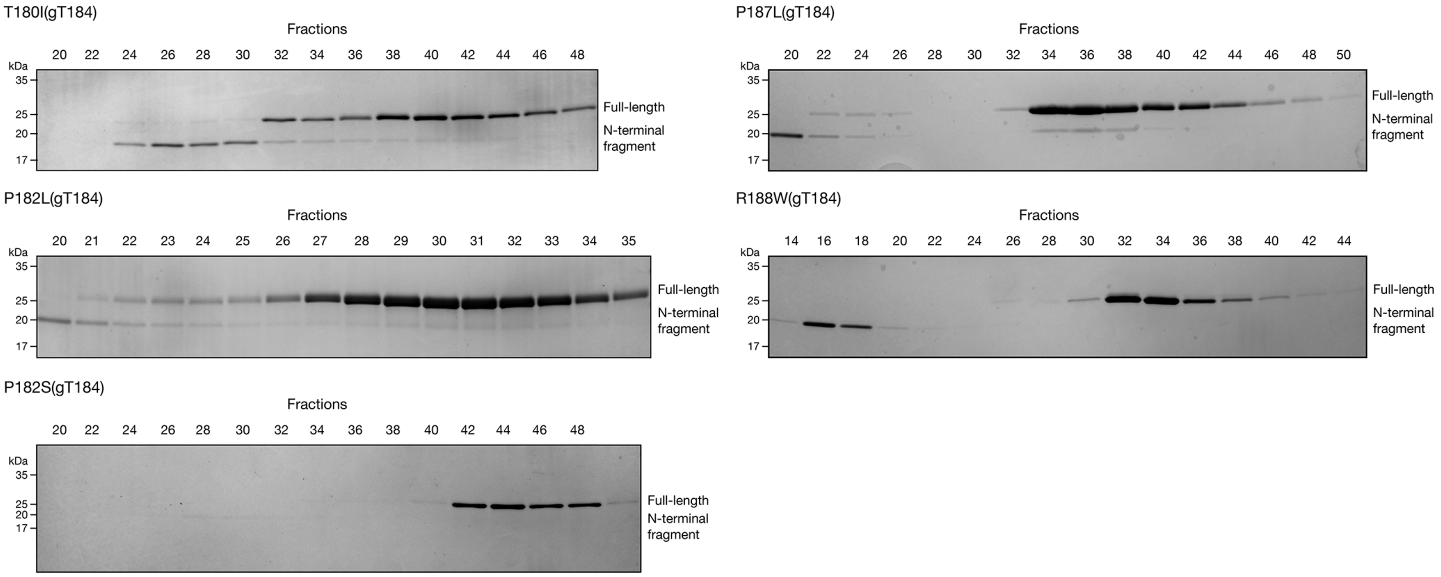

Figure S1. Purification of semisynthetic HSP27 proteins by anion exchange. Fractions from anion-exchange chromatography were analyzed by SDS-PAGE and Coomassie staining. Fractions with >95% pure full-length protein were pooled.

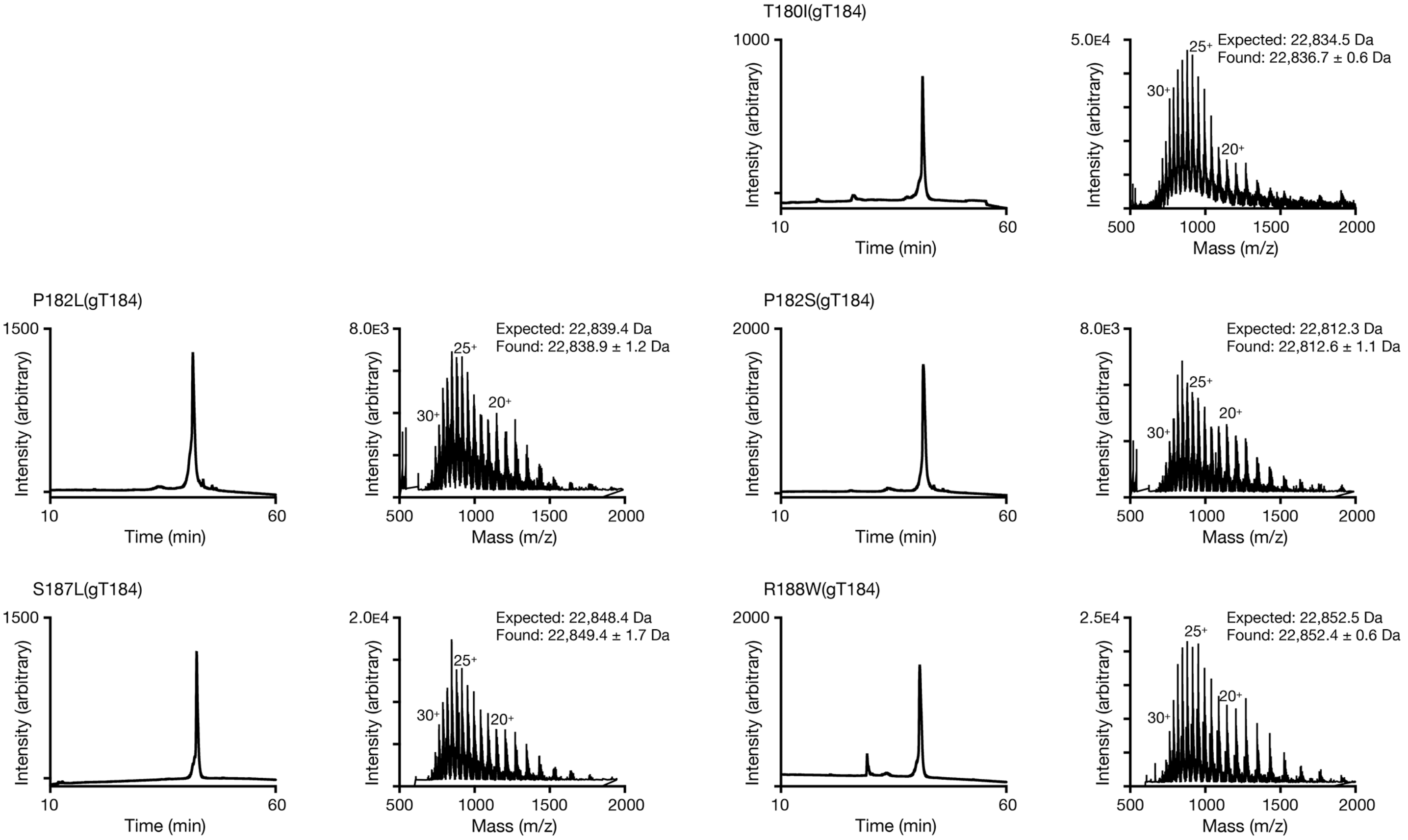

Figure S2. Characterization of O-GlcNAc modified proteins. RP-HPLC traces and ESI-MS characterization of the indicated O-GlcNAc-modified HSP27 proteins.

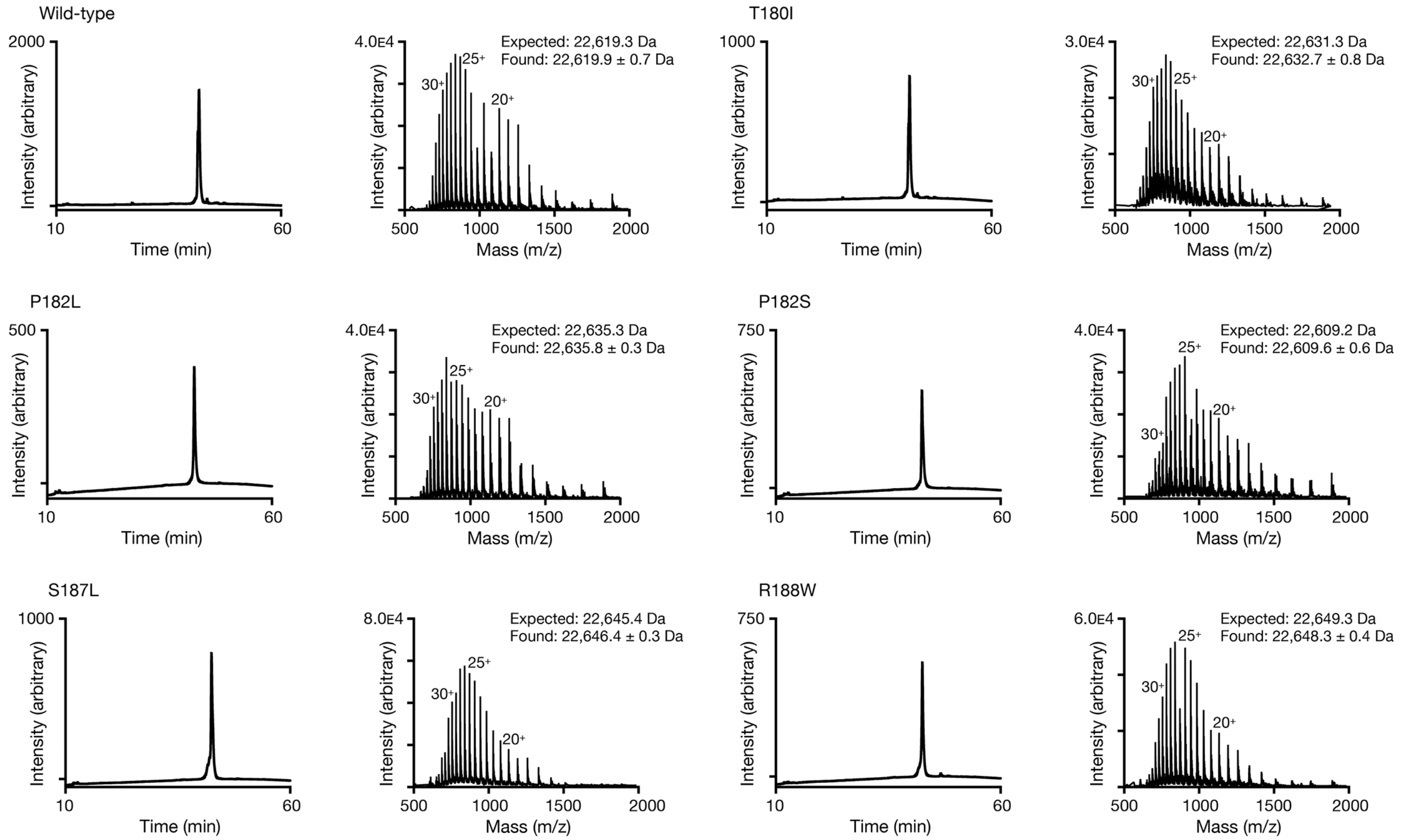

Figure S3. Characterization of unmodified proteins. RP-HPLC traces and ESI-MS characterization of the indicated recombinant HSP27 proteins.

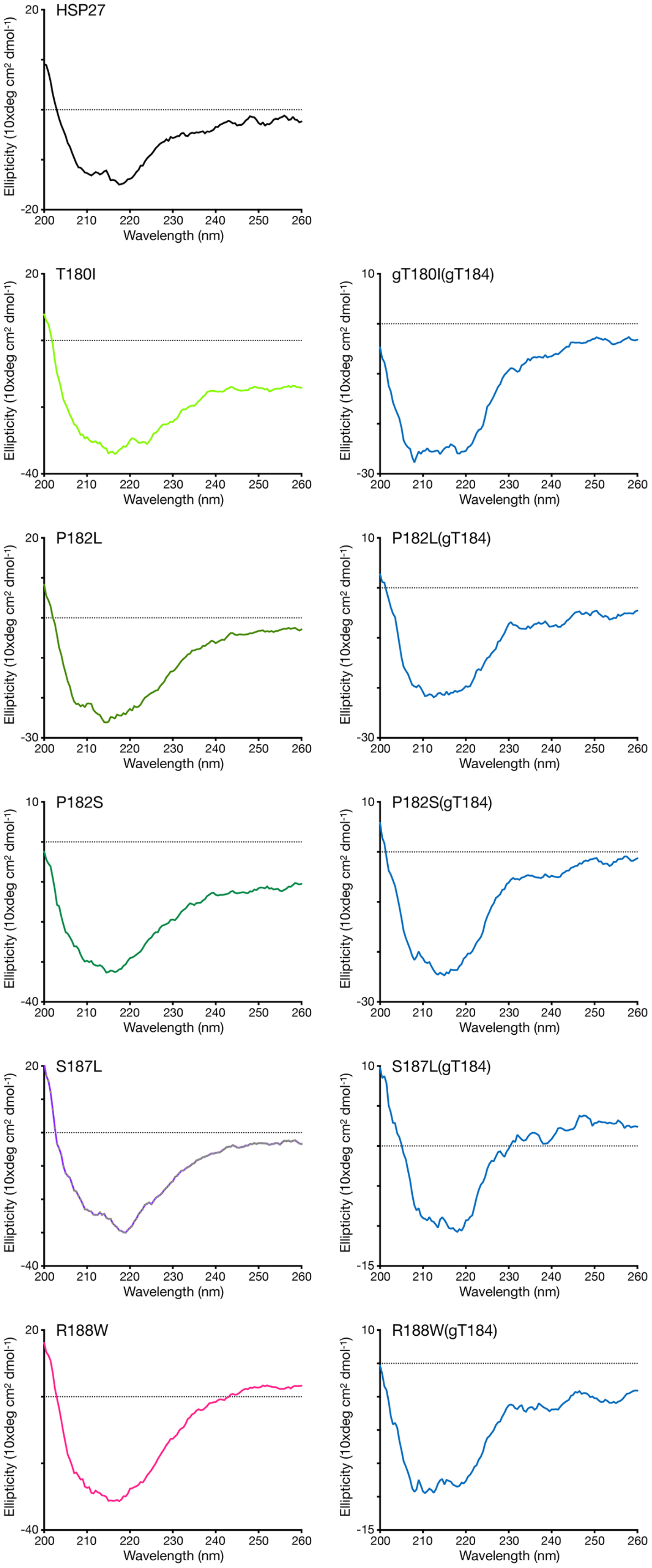

Figure S4. Circular dichroism (CD) spectra of all proteins. CD profiles of the recombinant and synthetic proteins.

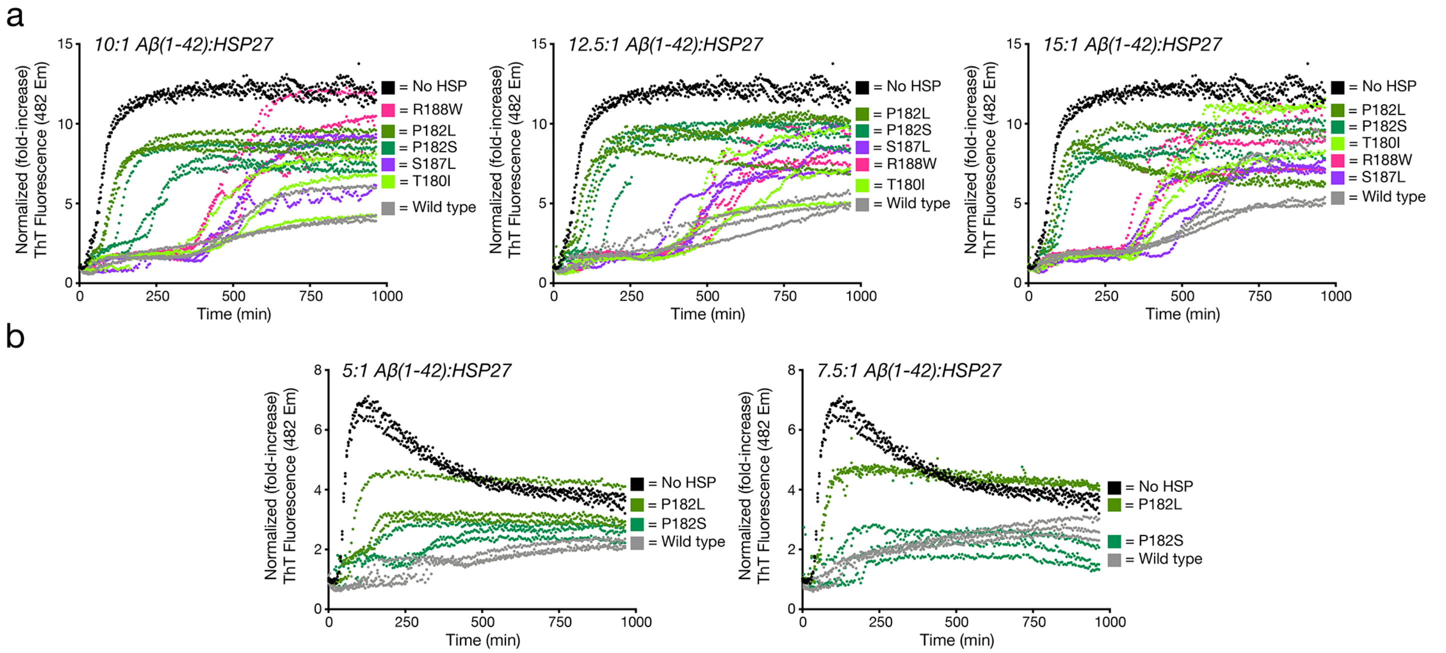

Figure S5. CMT2 mutants of HSP27 show different levels of compromised chaperone activity. Aβ(1-42) alone (10 μM) or in the presence of the indicated ratios of HSP27 proteins was subjected to aggregation conditions (agitation at 37 °C in a plate reader). Every 5 min, ThT fluorescence (λ_{ex} = 450 nm, λ_{em} = 482 nm).
